# Bacteriocin prediction through cross-validation-based and hypergraph-based feature evaluation approaches

**DOI:** 10.3389/fbinf.2025.1694009

**Published:** 2025-11-25

**Authors:** Suraiya Akhter, John H. Miller

**Affiliations:** 1 School of Electrical Engineering and Computer Science, Washington State University, Pullman, WA, United States; 2 School of Engineering and Applied Sciences, Washington State University Tri-Cities, Richland, WA, United States; 3 School of Business and Technology, Emporia State University, Emporia, KS, United States

**Keywords:** antimicrobial resistance, antimicrobial peptides, bacteriocin prediction, feature selection, machine learning, Shapley additive explanations, web application

## Abstract

Bacteriocins offer a promising solution to antibiotic resistance, possessing the ability to target a wide range of bacteria with precision. Thus, there is an urgent need for a computational model to predict new bacteriocins and aid in drug development. This work centers on constructing web-based predictive models using the XGBoost machine learning algorithm, based on the physicochemical properties, structural characteristics, and sequence profiles of protein sequences. We employed correlation analyses, cross-validation, and hypergraph-based techniques to select features. Cross-validated feature selection (CVFS) partitions the dataset, selects features within each partition, and identifies common features, ensuring representativeness. On the contrary, hypergraph-based feature evaluation (HFE) focuses on minimizing hypergraph cut conductance, leveraging higher-order data relationships to precisely utilize information regarding feature and sample correlations. The XGBoost models were built using the selected features obtained from these two feature evaluation methods. We also analyzed the feature contributions directly from the best model using SHapley Additive exPlanations (SHAP). Our HFE-based approach achieved 99.11% accuracy and an AUC of 0.9974 on the test data, overall outperforming the CVFS-based feature evaluation method and yielding results comparable to existing approaches. The most influential features are related to solvent accessibility for buried residues, followed by the composition of cysteine. Our web application, accessible at https://shiny.tricities.wsu.edu/bacteriocin-prediction/, offers prediction results, probability scores, and SHAP plots using both cross-validation- and hypergraph-based methods, along with previously implemented approaches for feature selection.

## Introduction

Antibiotics have been extensively utilized in animal husbandry and food processing to combat pathogens and extend shelf life, yet their usage has precipitated concerning consequences including bacterial resistance and the dissemination of antibiotic resistance genes (Braïek et al., 2018; Meade et al., 2020; Ren et al., 2022). This has prompted a shift towards natural alternatives, driven further by consumer preferences for additive-free, healthy foods (Khodaei and Sh, 2018). Consequently, there is a growing interest in exploring alternative antibacterial agents to control foodborne pathogens. Bacteriocins, proteins synthesized by bacteria, have emerged as promising antimicrobial agents due to their effectiveness against various microbes, including genetically similar strains (Meade et al., 2020). They offer advantages such as high efficacy, low toxicity, and minimal residue production, making them attractive substitutes for conventional antibiotics (Riley and Wertz, 2002; Hamid and Friedberg, 2017; Fields et al., 2020). Despite their potential, identifying and characterizing bacteriocins pose challenges. While conventional techniques such as screening assays, chromatography, and mass spectrometry are employed for this task (Zendo et al., 2008; Zhang et al., 2018; Desiderato et al., 2021), they frequently demonstrate to be lengthy, laborious, and expensive, possibly disregarding the breadth and originality of bacteriocins within intricate microbial populations (Perez et al., 2014).

To overcome constraints in identifying bacteriocins, computational methods like BLASTP are employed to predict them by recognizing patterns or motifs in bacteriocin sequences (Boratyn et al., 2013). Additional tools include BACTIBASE, which integrates microbial data from PubMed alongside protein examination utilities for the characterization of bacteriocins (Hammami et al., 2010), and BAGEL, which categorizes bacteriocin sequences based on homology (Van Heel et al., 2013; van Heel et al., 2018), each upholding repositories of validated bacteriocin sequences. Despite their utility, these methods rely on sequence alignment and may struggle with novel or highly diverse bacteriocins. antiSMASH, a different tool, utilizes hidden Markov models in conjunction with BLAST searches across a database of bacteriocin biosynthetic gene clusters to uncover potential clusters (Weber et al., 2015). While some tools like BOA aim to address bacteriocin diversity, they still rely on homology-based identification, limiting their ability to detect highly dissimilar bacteriocins lacking conserved context genes (Morton et al., 2015).

Machine learning algorithms offer a distinct approach from traditional sequence matching methods in bacteriocin prediction, enabling the detection of patterns and characteristics beyond mere similarity to known sequences. These algorithms can analyze physicochemical properties, sequence profiles, and secondary structures to uncover novel bacteriocins with significant dissimilarities. Recent advancements include the utilization of *k*-mer features and word embedding techniques, as presented in the studies by Mikolov et al. (Mikolov et al., 2013) and Hamid and Friedberg (Hamid and Friedberg, 2019). Furthermore, the RMSCNN technique, based on convolutional neural networks (CNNs), has been developed for bacteriocin prediction (Cui et al., 2021). Despite these advancements, existing methods overlook the importance of analyzing both primary and secondary peptide structures and lack feature evaluation mechanisms. To address these limitations, recently we unveiled BaPreS, a machine learning-based software tool, and BPAGS, a machine learning-based web application, which employ support vector machines (SVMs) and feature evaluation techniques such as *t*-tests, genetic algorithm and alternating decision tree to precisely identify novel bacteriocins (Akhter and Miller, 2023a; Akhter and Miller, 2023b).

In this work, our objective was to create web-based predictive models utilizing the XGBoost machine learning algorithm (Chen and Guestrin, 2016), incorporating physicochemical features, sequence profiles and structural properties of bacteriocin and non-bacteriocin sequences chosen through correlation analysis followed by sophisticated techniques like cross-validation (Yang and Wu, 2023) and hypergraph-based methods (Misiorek and Janowski, 2023). Subsequently, we evaluated the prediction performance of the models and examined the influence or significance of these selected features within the models using SHapley Additive exPlanations (SHAP) (Lundberg et al., 2020). We integrated cross-validated feature selection (CVFS) and hypergraph-based feature evaluation (HFE) methods, along with corresponding SHAP analyses, into our existing web-based tool available at https://shiny.tricities.wsu.edu/bacteriocin-prediction/ (Akhter and Miller, 2023b). This enhancement allows users to choose CVFS and HFE techniques, along with selecting features based on alternating decision tree, genetic algorithm, linear SVC, or *t*-test methods to obtain precise prediction results and probability scores by cross-checking with different feature evaluation methods. Our web tool also automatically generates the necessary features from user-supplied protein sequences. Users have the capability to concurrently assess numerous sequences and incorporate fresh data, thereby boosting the capacity of the predictive models embedded within the web application.

## Methods

**FIGURE 1:**
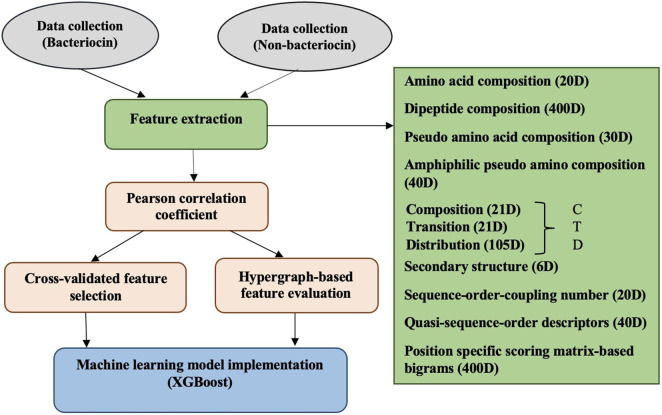
Illustrating the procedure for bacteriocin detection.

The complete process of our approach is illustrated in Figure 1. It involves gathering protein datasets for both bacteriocin (positive) and non-bacteriocins (negative), creating possible candidate features, employing feature assessment methods, applying the Pearson correlation coefficient, followed by CVFS and HFE techniques to remove the least significant and irrelevant features. Subsequently, selected feature sets are used to construct machine learning models for assessing prediction performance.

### Datasets

The datasets utilized in this work align with those employed in the creation of our previously released software and web applications (Akhter and Miller, 2023a; Akhter and Miller, 2023b). Experimentally validated bacteriocin sequences were retrieved from two publicly available databases, BAGEL (Van Heel et al., 2013; van Heel et al., 2018) and BACTIBASE (Hammami et al., 2010), both of which curate bacteriocins with confirmed antimicrobial activity. Non-bacteriocin protein sequences were obtained from the RMSCNN dataset (Cui et al., 2021), which includes bacterial proteins not associated with bacteriocin function. Initially, a total of 483 positive and 500 negative protein sequences were collected. To eliminate redundancy and minimize potential sequence-level bias, the CD-HIT clustering algorithm (Fu et al., 2012) was applied with a 90% sequence similarity threshold, thereby removing duplicate or highly similar entries. Although more stringent thresholds can further reduce redundancy, a 90% cutoff was chosen to preserve sequence diversity within the bacteriocin family, as novel bacteriocins often share considerable similarity with known ones (Darbandi et al., 2022; Lertampaiporn et al., 2021; Mesa-Pereira et al., 2018; Daw and Falkiner, 1996). After filtering, 283 unique positive and 497 unique negative sequences were retained.

To address the challenge of imbalanced data, random sampling was employed, resulting in a reduction of negative sequences to 283, thereby attaining equilibrium between these two groups of the sequences. For training purposes, 80% of the data set was assigned, leaving the remaining 20% for testing. The Supplementary Material contains both the training and testing datasets.

### Features

Constructing robust predictive machine learning models depends greatly on identifying and extracting potential attributes. In our work, we developed various sets of features to encompass diverse aspects of protein sequences. These comprised a 20-dimensional amino acid composition (AAC), a 400-dimensional dipeptide composition (DC), a 30-dimensional pseudo amino acid composition (PseAAC), and a 40-dimensional amphiphilic pseudo amino acid composition (APseAAC). Moreover, we applied the composition/transition/distribution (CTD) model (Dubchak et al., 1995), yielding 147-dimensional attribute sets that account for a range of physicochemical characteristics of amino acids. Furthermore, we devised 6-dimensional feature sets to illustrate the secondary structure (SS) nuances such as α-helix, β-strand, and γ-coil within individual protein sequences. We employed the amino acid distance matrix to generate 20-dimensional sequence-order-coupling number (SOCN) feature sets and 40-dimensional quasi-sequence-order (QSO) feature sets for every sequence (Xiao et al., 2015). We also employed the position-specific scoring matrix (PSSM) to extract features reflecting evolutionary trends, resulting in a 400-dimensional attribute set for each sequence by calculating transition scores between adjacent amino acids derived from the PSSM (S et al., 2016; Mohammadi et al., 2022). Detailed elucidations of these feature sets are provided in our developed BaPreS software tool (Akhter and Miller, 2023a) and BPAGS web application (Akhter and Miller, 2023b). In total, 1,103 features were considered as candidates.

### Feature assessment

To ensure the efficacy of a predictive model, it is essential to eliminate irrelevant features before building the model. Our initial step involved examining the correlation among these features using the Pearson correlation coefficient, employing a methodology akin to that utilized in our earlier implemented BaPreS (Akhter and Miller, 2023a) and BPAGS (Akhter and Miller, 2023b) tools. To prevent any inadvertent sharing of information between training and testing datasets, our focus remained solely on features within the training data. Model hyperparameters were optimized using grid search exclusively within the training process, and final evaluations were performed on independent test data. These safeguards ensured that the model’s performance metrics were unbiased and reflected genuine generalization rather than overfitting. When two features exhibited a high correlation (≥0.9), one was retained while the other was discarded. This choice led to a reduction in the number of features from 1,103 to 602. The Supplementary Material, specifically Supplementary Table S1, provides a comprehensive list of reduced features utilized in our study. These features are represented by abbreviations such as “aac,” “dipep,” “pseudo,” “amphipseudo,” “comp,” “tran,” “dist,” “ss,” “qso,” and “pssm,” which correspond to amino acid composition (AAC), dipeptide composition (DC), pseudo amino acid composition (PseAAC), amphiphilic pseudo amino acid composition (APseAAC), composition (CTD), transition (CTD), distribution (CTD), secondary structure (SS), quasi-sequence-order (QSO), and position-specific scoring matrix (PSSM)-based features, respectively. These reduced features have been comprehensively clarified in our previously published work, BPAGS (Akhter and Miller, 2023b).

**FIGURE 2:**
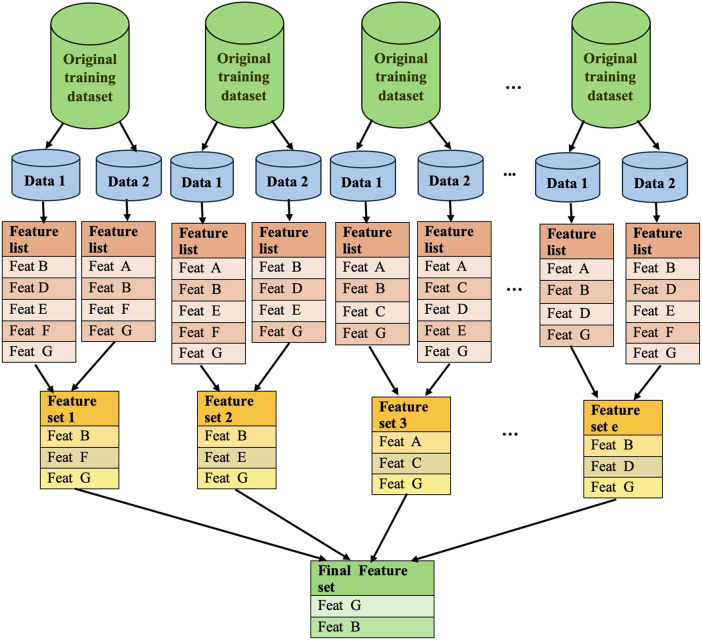
Depicting the procedure of selecting features using the CVFS approach.

We opted to employ the CVFS technique (Yang and Wu, 2023) along with a hypergraph based method (Misiorek and Janowski, 2023) to further distill features obtained via Pearson’s correlation analysis. The overall workflow of the CVFS algorithm is illustrated in Figure 2. In this approach, the dataset was randomly divided into *c* non-overlapping subsets. An XGBoost model was trained independently on each subset to estimate feature importance based on model-gain values, with hyperparameters optimized via grid search to ensure reproducible performance across subsets. The top-ranked features from each subset were intersected to obtain the features consistently selected across all partitions. This entire procedure was repeated *e* times with different random splits, generating *e* intersected feature sets. A feature was included in the final selection if it appeared in at least *p* × 100% of these intersection sets.

In this study, we used *c* = 2 and *e* = 5 and 10 to maintain sufficient training size while ensuring stability of intersection-based selection under a limited sample size. The parameter *p* controlled the stability requirement, and features retained in the majority of intersections were included in the final set. These settings provided a balance between computational efficiency and robustness, as confirmed by consistent feature rankings and predictive metrics across multiple repetitions. This repeated procedure ensured that only stable and reproducible features contributing meaningfully to model performance were retained. The number of features in the subsets obtained with different combinations of *c*, *e*, and *p* values is shown in Table 1, and the full list of selected features is provided in Supplementary Tables S2–S5 (Supplementary Material).

**TABLE 1:** Number of features, MCC, accuracy, precision, recall, F1 and AUC values for testing data for various feature subsets. The most effective models for CVFS and HFE feature sets are highlighted in bold.

Feature evaluation algorithm	Configuration	Number of features	$Test_{Mcc}$	$Test_{Acc}$	$Test_{Precision}$	$Test_{Recall}$	$Test_{F1}$	$Test_{AUC}$
CVFS	**(*c* = 2, *e* = 5, *p* = 0.4)**	**27**	**0.9823**	**0.9911**	**1.0000**	**0.9821**	**0.9910**	**0.9962**
	(*c* **=** 2, *e* **=** 5, *p* = 0.6)	16	0.9466	0.9732	0.9649	0.9821	0.9735	0.9904
	(*c* = 2, *e* = 5, *p* = 0.8)	10	0.9466	0.9732	0.9649	0.9821	0.9735	0.9888
	(*c* = 2, *e* = 10, *p* = 0.4)	24	0.9823	0.9911	1.0000	0.9821	0.9910	0.9924
HFE Bin = 5	*β* = 15	90	0.9649	0.9821	1.0000	0.9643	0.9818	0.9965
	*β* = 30	181	0.9823	0.9911	1.0000	0.9821	0.9910	0.9952
	***β* = 50**	**301**	**0.9823**	**0.9911**	**1.0000**	**0.9821**	**0.9910**	**0.9974**
HFE **Bin = 10**	*β* = 15	90	0.9465	0.9732	0.9649	0.9821	0.9735	0.9904
	*β* **= 30**	**181**	**0.9823**	**0.9911**	**1.0000**	**0.9821**	**0.9910**	**0.9974**
	*β* = 50	301	0.9823	0.9911	1.0000	0.9821	0.9910	0.9949

Legend: $Test_{acc}$ : Accuracy on the testing dataset.

$Test_{precision}$ : Precision on the testing dataset.

$Test_{recall}$ : Recall on the testing dataset.

${Test}_{F1}$ : F1 score on the testing dataset.

$Test_{AUC}$ : AUC on the testing dataset.

*c*: Number of disjoint sub-parts.

*e*: Number of repeated runs.

*p*: Proportions of repeated runs for extracting common features.

*β*: percentage of selected features.

**FIGURE 3:**
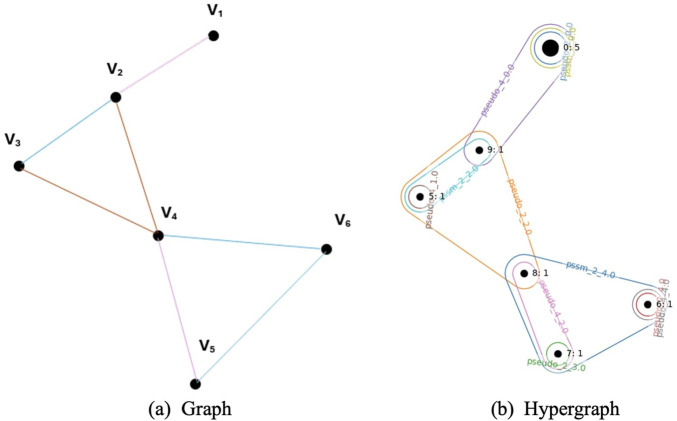
**(a)** A standard graph where each edge connects two vertices. **(b)** A hypergraph where each hyperedge can connect multiple vertices.

The HFE method evaluates feature importance by modeling the dataset as a hypergraph that captures higher order relationships among feature values and class labels. In contrast to a conventional graph G = (*V*, *E*) where an edge connects two vertices, a hypergraph *H* = (*V*, *E*) allows a hyperedge to connect multiple vertices. In our setting, vertices represent data samples and hyperedges represent groups of samples that share a discretized feature value. Continuous features were discretized into uniform bins, and each bin defined a hyperedge linking all samples that fell within the corresponding value range. Two additional hyperedges were created for class labels, ensuring that positive and negative classes were explicitly encoded. Figure 3 illustrates the difference between a graph and a hypergraph.

Once the hypergraph was constructed, feature relevance was assessed using the hypergraph cut conductance minimization framework of Misiorek and Janowski (Misiorek and Janowski, 2023). This approach employs lazy random walks across the hypergraph to estimate how likely it is for a walk starting from one class to remain in the same class or transition into the opposite class. At each step, an incident hyperedge is selected with probability proportional to its weight, and then a vertex within that hyperedge is selected with probability proportional to its vertex weight. From this process, three importance ratings are derived: *R*_*1*_, which measures preference toward preserving minority class labels; *R*_*2*_, which measures preference toward preserving majority class labels; and *R*_*0*_, which measures the ability to separate samples of different classes. Together, these hypergraph-based ratings capture the contribution of feature values to class separability.

For each feature, hypergraph importance ratings across all bins were aggregated to compute a single feature-level score. Features were then ranked according to these scores, and the top *z* = *β* × *m* were retained, where *m* is the total number of features and *β* is a user-defined proportion. Multiple values of *β* were tested for both bin sizes. In our case, we considered bin = 5 and bin = 10, as these values provided a practical balance between capturing sufficient variability in the feature distributions and avoiding sparsity in the hypergraph representation (see Supplementary Figure S1 in the Supplementary Material). The parameter β was tested at 15%, 30%, and 50%, controlling the final feature budget and enabling the evaluation of compact, moderate, and extended feature subsets. These parameter choices were empirically determined to achieve stable rankings of important features and consistent model performance across configurations, confirming the robustness of the approach. The numbers of features selected for different *β* and bin settings are presented in Table 1, and the corresponding reduced feature lists are provided in Supplementary Tables S6 and S7 (Supplementary Material).

### Web application

**FIGURE 4:**
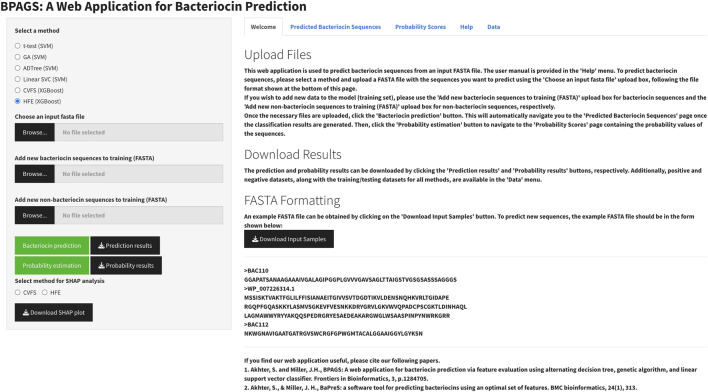
The web application for bacteriocin prediction.

The CVFS and HFE methods were integrated into the web application along with previously implemented feature evaluation approaches (Akhter and Miller, 2023b), as illustrated in Figure 4. This machine learning-based web tool autonomously generates features for user-provided sequences, yielding classification and probability results. Details on data upload, binary classification, and probability estimation are outlined within the web application, and a user can download it from the web application. Users can download necessary files and augment training data with new protein sequences, enhancing prediction accuracy. The updated web application now provides users the facility of downloading the SHAP plot to inspect the impact of the top 10 features on the prediction outcomes. The web server can be publicly accessible at https://shiny.tricities.wsu.edu/bacteriocin-prediction/.

### Performance measurement

Evaluation of a model’s predictive accuracy on the testing dataset was conducted using Equations 1–5, where TP, TN, FP, and FN denote true positives, true negatives, false positives, and false negatives, respectively. To assess classifier effectiveness, accuracy assesses the proportion of accurately categorized instances compared to the total instances within the dataset. We utilized the Matthews correlation coefficient (MCC), a metric ranging from −1 to +1, with higher values indicating superior prediction capabilities.

$$Test_{Acc}=\frac{TP+TN}{TP+TN+FP+FN}$$ (1)

$$Test_{MCC}=\frac{TP\times TN-FP\times FN}{\sqrt{\left(TP+FP\right)\left(TP+FN\right)\left(TN+FP\right)\left(TN+FN\right)}}$$ (2)

$$Test_{recall}=\frac{TP}{TP+FN}$$ (3)

$$Test_{precision}=\frac{TP}{TP+FP}$$ (4)

$${Test}_{F1}=2\times \frac{\left({Test}_{precision}\times {Test}_{recall}\right)}{\left({Test}_{precision}+{Test}_{recall}\right)}$$ (5)

We also calculated recall and precision. Recall measures the proportion of correctly identified true positive instances, while precision evaluates the fraction of accurate positive predictions. The F1 score, a metric that accounts for both precision and recall, computes their harmonic mean, thus presenting a well-rounded assessment of the model’s efficacy. Furthermore, we determined the Area Under the Curve (AUC) to appraise the efficacy of binary classification models. A greater AUC value signifies better performance, where 1 denotes perfection and 0.5 indicates random chance.

### Code and data availability

The complete set of experimental data and accompanying scripts is available at https://github.com/suraiya14/cvfs_hfe.

## Results

After downsizing the feature collection through two distinct feature assessment techniques, we constructed separate predictive models utilizing the chosen features through the XGBoost (Chen and Guestrin, 2016) machine learning technique. By employing the SHAP (Shapley Additive Explanations) approach (Lundberg et al., 2020), we evaluated the significance of features and their contributions to the XGBoost models. SHAP values quantify the additional influence of each feature on the forecasts generated by the machine learning model.

### Model performance

We developed an XGBoost model by training it on various feature subsets derived from CVFS and HFE analyses alongside the training dataset. Table 1 elaborates on the evaluation of XGBoost models across CVFS and HFE-based feature subsets, while Supplementary Figure S2 (Supplementary Material) illustrates the confusion matrices for all reduced feature sets. Overall, we obtained better prediction results for the HFE (bin = 10, *β* = 30) feature set compared to the CVFS (*c* = 2, *e* = 5, *p* = 0.4) feature set, with our best model able to identify 55 protein sequences.

As mentioned earlier, the top-performing machine-learning outcome was achieved through implementing the XGBoost model, utilizing HFE method with bin = 10 and *β* = 30 parameters. The HFE selected 181 features from a pool of 701, predominantly focusing on dipeptide composition and distribution features. For detailed insight into the chosen features on the training data, please refer to Supplementary Table S7 provided in the Supplementary Material.

### Feature contribution analysis

**FIGURE 5:**
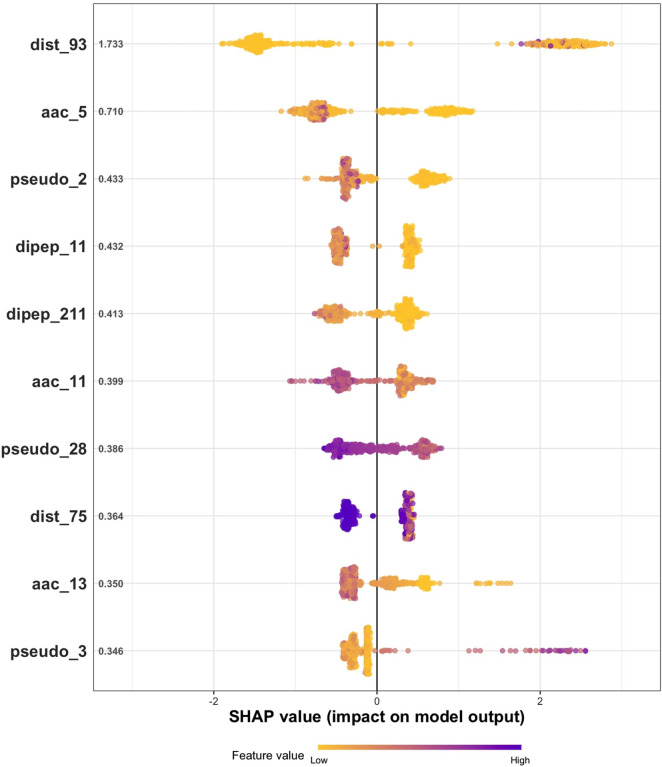
Feature importance (SHAP values) obtained from the XGBoost model for predicting bacteriocins.

Figure 5 displays the ranking of the top 10 features based on their mean SHAP values for predicting bacteriocins using the best XGBoost model with hypergraph (bin = 10, *β* = 30) reduced feature sets. Each point on the plot represents a protein sequence, with overlapping points visualized through jittering to indicate their frequency. The *x*-axis indicates the influence of features on the model’s output, which is either a prediction of 1 (bacteriocin) or 0 (non-bacteriocin). The *y*-axis shows the mean |SHAP| values of the features. The color bar at the bottom of the figure represents the value of features where yellow and purple correspond to low and high values, respectively. The features in the plot are in ascending of importance determined based on the mean |SHAP| of the features. A detailed description of the features can be found in our previous study and in protr/ProtrWeb (Akhter and Miller, 2023b; Xiao et al., 2015).

To further interpret the SHAP results, we examined the biological relevance of the most influential features. The top-ranked feature, *dist_93*, is derived from the CTD model (Dubchak et al., 1995) and represents the solvent-accessibility descriptor corresponding to group 1 (buried residues). Solvent-accessibility descriptors are widely used in peptide and protein classification tasks because residue exposure and burial strongly correlate with stability and function (Tien et al., 2013; Chang et al., 2008). In our case, higher *dist_93* values indicate a greater proportion of buried hydrophobic residues, which are characteristic of bacteriocins that form compact, stable cores enabling membrane interaction. The second most significant feature, *aac_5*, corresponds to the amino-acid composition of cysteine residues. Inspection of the SHAP color gradient indicates that higher *aac_5* values tend to yield negative SHAP values, shifting predictions toward non-bacteriocin, whereas lower *aac_5* values show a broader spread with many positive SHAP contributions. Thus, increased cysteine content generally decreases the probability of bacteriocin classification, while reduced cysteine content is associated with a higher probability on average, although this effect is not absolute and depends on the context of other features. This trend is consistent with reports that many bacteriocins and related antimicrobial peptides achieve activity without relying on disulfide bonds, whereas cysteine-rich peptides can adopt distinct stabilized conformations (Gongora-Benitez et al., 2014; Ma et al., 2024).

Other ranked features (pseudo_2, dipep_11, dipep_211, aac_11, pseudo_28, dist_75, aac_13, and pseudo_3) also contributed modestly to the model predictions, as reflected by their moderate mean SHAP values and balanced distribution of positive and negative impacts in the plot. These features represent additional sequence- and composition-based descriptors that provide complementary information to the most influential variables. Together, these observations show that the model primarily relies on solvent-accessibility and cysteine-composition signals, with secondary contributions from sequence-order and compositional descriptors. The complete SHAP importance plot for all features is provided in Supplementary Figure S3 (Supplementary Material).

**FIGURE 6:**
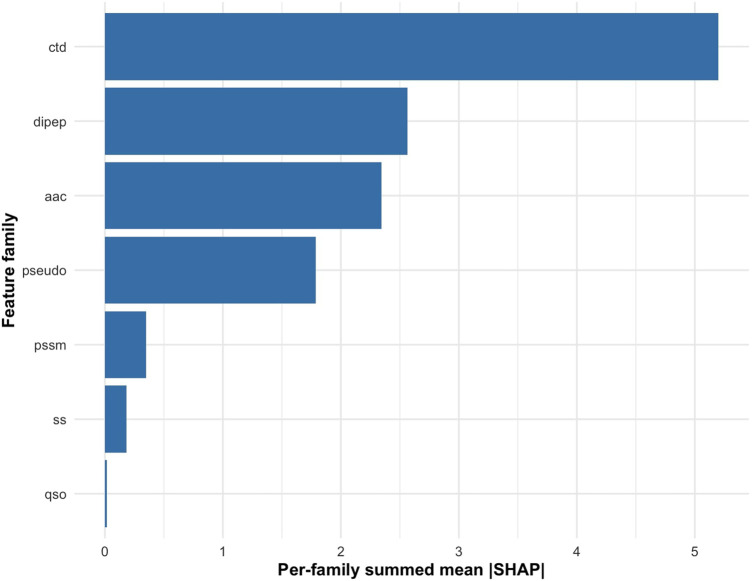
Summed SHAP importance of different feature families (CTD, DC, AAC, PseAAC, PSSM, SS, and QSO) showing their relative contributions to bacteriocin prediction.

To compare the relative importance of different feature families, we aggregated the mean absolute SHAP values across all features within each descriptor category. The results (Figure 6) show that CTD-based features contributed the greatest overall importance, highlighting the role of residue distribution and physicochemical composition patterns in bacteriocin classification. DC and AAC features provided substantial contributions, reflecting the influence of short-range residue pair frequencies and global compositional characteristics. PseAAC descriptors showed moderate contributions, while features derived from PSSM, SS, and QSO descriptors contributed relatively little. Overall, these results indicate that compositional and distribution-based properties are the main determinants guiding model predictions, whereas sequence-order and structural descriptors provide complementary but less influential information.

These findings are consistent with established principles of antimicrobial peptide structure and function (Daw and Falkiner, 1996; Rost and Sander, 1993; Drider et al., 2006; Cotter et al., 2013; Jenssen et al., 2006; Brogden, 2005; Wimley, 2010; Nguyen et al., 2011; Fimland et al., 2005; Oman and Van Der Donk, 2010; Yang et al., 2014). Solvent accessibility influences peptide folding, hydrophobic-core stability, and membrane-binding potential. Many bacteriocins form amphipathic helices or hydrophobic cores that promote membrane insertion and pore formation. Conversely, cysteine composition governs disulfide-bond formation and structural stabilization. The observed negative association between cysteine content and bacteriocin probability supports prior evidence that many active bacteriocins function effectively without disulfide bridges. Although wet-lab validation lies beyond the present computational scope, all predictions and SHAP-based interpretations are available through our web platform (https://shiny.tricities.wsu.edu/bacteriocin-prediction/), providing a transparent framework for future experimental verification.

### Performance comparison

The prediction performance of the XGBoost models built using CVFS-reduced feature sets and HFE-reduced feature sets was compared with the deep learning method RMSCNN (Cui et al., 2021), as well as our previously introduced BaPreS and BPAGS tools (Akhter and Miller, 2023a; Akhter and Miller, 2023b). RMSCNN, developed specifically for the detection of marine microbial bacteriocins through Convolutional Neural Networks (CNN), transforms protein sequences into numeric formats to facilitate feature acquisition and prediction. BaPreS and BPAGS automate feature generation and selection through correlation and *t*-test analyses, alternating decision tree, and genetic algorithm, employing Support Vector Machines (SVMs) for prediction. All models were tuned using the parameter settings described in the original studies to ensure reproducibility and fair comparison. Our XGBoost models demonstrated superior performance compared to RMSCNN and BaPreS, while providing comparable prediction results to BPAGS (see Table 2).

**TABLE 2:** Evaluation of the efficacy of models/tools in predicting bacteriocins.

Method/tool	$Test_{acc}$	$Test_{precision}$	$Test_{recall}$	${Test}_{F1}$	${Test}_{AUC}$
RMSCNN	0.9375	0.9623	0.9107	0.9358	0.9818
BaPreS	0.9554	0.9636	0.9464	0.9550	0.9879
BPAGS (ADTree)	0.9911	0.9825	1.0000	0.9912	0.9984
BPAGS (GA)	0.9643	0.9643	0.9643	0.9643	0.9968
BPAGS (Linear SVC)	0.9732	0.9818	0.9643	0.9730	0.9990
CVFS (*c* = 2, *e* = 5, *p* = 0.4)	0.9911	1.0000	0.9821	0.9910	0.9962
HFE (bin = 5, *β* = 30)	0.9911	1.0000	0.9821	0.9910	0.9974

Legend: $Test_{acc}$ : Accuracy on the testing dataset.

$Test_{precision}$ : Precision on the testing dataset.

$Test_{recall}$ : Recall on the testing dataset.

${Test}_{F1}$ : F1 score on the testing dataset.

$Test_{AUC}$ : AUC on the testing dataset.

## Discussion

The pursuit of new bacteriocins is essential for advancing the creation of fresh antibiotic treatments to counter the escalating threat of antibiotic resistance. This work introduces web-based predictive models aimed at identifying novel bacteriocins. Our approach involves extracting diverse features from primary and secondary attributes of protein sequences, alongside sequence profiles. These features are then subjected to analysis using the Pearson correlation coefficient, followed by CVFS and HFE feature evaluations. Subsequently, we employed the XGBoost machine-learning algorithm using the selected feature sets. Our findings indicate that XGBoost demonstrates superior predictive capabilities, particularly when using the HFE-reduced feature set.

Solvent accessibility (CTD model) was identified as the most influential feature, followed by cysteine composition (AAC). The solvent accessibility influences various aspects of bacteriocins, including their interaction with target bacterial membranes and their stability in the extracellular environment, and cysteine residues have been reported as important structural determinants in certain bacteriocins such as bactofencin A (Benítez-Chao et al., 2021; O’Connor et al., 2018). Collectively, these analyses demonstrate that solvent-accessibility, compositional, and distribution-based properties are the dominant determinants guiding model predictions.

The efficacy of our most proficient model was evaluated by comparing it with both deep-learning methods and tools we had previously created. The findings indicate that XGBoost demonstrated comparable or improved performance in comparison. Our web application integrates both CVFS and HFE for feature evaluation, incorporating all necessary programs to automatically generate an optimal feature set. Users can now utilize CVFS and HFE alongside existing methods to predict bacteriocin presence in unseen testing data. Additionally, they can augment the training data with new bacteriocin and non-bacteriocin sequences and perform SHAP analyses, enhancing the predictive capability of the web tool.

This study has several limitations. Presently, our model proficiently discerns singular bacteriocin protein sequences, and our goal is to improve its capability to identify protein clusters such as tailocins (bacteriocins resembling phage tails). Moreover, SHAP-based interpretations are correlational and not experimentally validated. Future *in vitro* work will be necessary to confirm the activity of newly predicted bacteriocins. The training and evaluation of the models were conducted on currently available bacteriocin and non-bacteriocin sequences, which may not fully represent the vast diversity of bacteriocins present in nature. We will maintain and update our machine-learning-based web application as more experimentally validated bacteriocin sequences become available. Further improvements will include the incorporation of granular molecular-component-based features and protein–protein-interaction-network-based features and the development of a robust feature-selection algorithm to enhance prediction accuracy.

## Data Availability

The complete set of experimental data and accompanying scripts is available at https://github.com/suraiya14/cvfs_hfe.

## References

[B1] Akhter S. Miller J. H. (2023a). BaPreS: a software tool for predicting bacteriocins using an optimal set of features. BMC Bioinforma. 24 (1), 313. 10.1186/s12859-023-05330-z 37592230 PMC10433575

[B2] Akhter S. Miller J. H. (2023b). BPAGS: a web application for bacteriocin prediction *via* feature evaluation using alternating decision tree, genetic algorithm, and linear support vector classifier. Front. Bioinforma. 3, 1284705. 10.3389/fbinf.2023.1284705 38268970 PMC10807691

[B3] Benítez-Chao D. F. León-Buitimea A. Lerma-Escalera J. A. Morones-Ramírez J. R. (2021). Bacteriocins: an overview of antimicrobial, toxicity, and biosafety assessment by *in vivo* models. Front. Microbiol. 12, 630695. 10.3389/fmicb.2021.630695 33935991 PMC8083986

[B4] Boratyn G. M. Camacho C. Cooper P. S. Coulouris G. Fong A. Ma N. (2013). BLAST: a more efficient report with usability improvements. Nucleic acids Res. 41 (W1), W29–W33. 10.1093/nar/gkt282 23609542 PMC3692093

[B5] Braïek O. B. Morandi S. Cremonesi P. Smaoui S. Hani K. Ghrairi T. (2018). Safety, potential biotechnological and probiotic properties of bacteriocinogenic Enterococcus lactis strains isolated from raw shrimps. Microb. Pathog. 117, 109–117. 10.1016/j.micpath.2018.02.021 29438718

[B6] Brogden K. A. (2005). Antimicrobial peptides: pore formers or metabolic inhibitors in bacteria? Nat. Rev. Microbiol. 3 (3), 238–250. 10.1038/nrmicro1098 15703760

[B7] Chang D. T.-H. Huang H.-Y. Syu Y.-T. Wu C.-P. (2008). Real value prediction of protein solvent accessibility using enhanced PSSM features. BMC Bioinforma. 9 (Suppl. 12), S12. 10.1186/1471-2105-9-s12-s12 19091011 PMC2638152

[B8] Chen T. Guestrin C. (2016). “Xgboost: a scalable tree boosting system,” in Proceedings of the 22nd acm sigkdd international conference on knowledge discovery and data mining.

[B9] Cotter P. D. Ross R. P. Hill C. (2013). Bacteriocins—A viable alternative to antibiotics? Nat. Rev. Microbiol. 11 (2), 95–105. 10.1038/nrmicro2937 23268227

[B10] Cui Z. Chen Z.-H. Zhang Q.-H. Gribova V. Filaretov V. F. Rmscnn H. D.-S. (2021). A random multi-scale convolutional neural network for marine microbial bacteriocins identification. IEEE/ACM Trans. Comput. Biol. Bioinforma. 19 (6), 3663–3672. 10.1109/TCBB.2021.3122183 34699364

[B11] Darbandi A. Asadi A. Mahdizade Ari M. Ohadi E. Talebi M. Halaj Zadeh M. (2022). Bacteriocins: properties and potential use as antimicrobials. J. Clin. Laboratory Analysis 36 (1), e24093. 10.1002/jcla.24093 34851542 PMC8761470

[B12] Daw M. A. Falkiner F. R. (1996). Bacteriocins: nature, function and structure. Micron 27 (6), 467–479. 10.1016/s0968-4328(96)00028-5 9168627

[B13] Desiderato C. K. Sachsenmaier S. Ovchinnikov K. V. Stohr J. Jacksch S. Desef D. N. (2021). Identification of potential probiotics producing bacteriocins active against Listeria monocytogenes by a combination of screening tools. Int. J. Mol. Sci. 22 (16), 8615. 10.3390/ijms22168615 34445321 PMC8395247

[B14] Drider D. Fimland G. Héchard Y. McMullen L. M. Prévost H. (2006). The continuing story of class IIa bacteriocins. Microbiol. Mol. Biol. Rev. 70 (2), 564–582. 10.1128/mmbr.00016-05 16760314 PMC1489543

[B15] Dubchak I. Muchnik I. Holbrook S. R. Kim S.-H. (1995). Prediction of protein folding class using global description of amino acid sequence. Proc. Natl. Acad. Sci. 92 (19), 8700–8704. 10.1073/pnas.92.19.8700 7568000 PMC41034

[B16] Fields F. R. Freed S. D. Carothers K. E. Hamid M. N. Hammers D. E. Ross J. N. (2020). Novel antimicrobial peptide discovery using machine learning and biophysical selection of minimal bacteriocin domains. Drug Dev. Res. 81 (1), 43–51. 10.1002/ddr.21601 31483516 PMC9202646

[B17] Fimland G. Johnsen L. Dalhus B. Nissen‐Meyer J. (2005). Pediocin‐like antimicrobial peptides (class IIa bacteriocins) and their immunity proteins: biosynthesis, structure, and mode of action. J. peptide Sci. official Publ. Eur. Peptide Soc. 11 (11), 688–696. 10.1002/psc.699 16059970

[B18] Fu L. Niu B. Zhu Z. Wu S. Li W. (2012). CD-HIT: accelerated for clustering the next-generation sequencing data. Bioinformatics 28 (23), 3150–3152. 10.1093/bioinformatics/bts565 23060610 PMC3516142

[B19] Gongora-Benitez M. Tulla-Puche J. Albericio F. (2014). Multifaceted roles of disulfide bonds. Peptides as therapeutics. Chem. Rev. 114 (2), 901–926. 10.1021/cr400031z 24446748

[B20] Hamid M. N. Friedberg I. (2017). “Bacteriocin detection with distributed biological sequence representation,” in ICML computational Biology workshop.

[B21] Hamid M.-N. Friedberg I. (2019). Identifying antimicrobial peptides using word embedding with deep recurrent neural networks. Bioinformatics 35 (12), 2009–2016. 10.1093/bioinformatics/bty937 30418485 PMC6581433

[B22] Hammami R. Zouhir A. Le Lay C. Ben Hamida J. Fliss I. (2010). BACTIBASE second release: a database and tool platform for bacteriocin characterization. Bmc Microbiol. 10 (1), 22–25. 10.1186/1471-2180-10-22 20105292 PMC2824694

[B23] Jenssen H. Hamill P. Hancock R. E. (2006). Peptide antimicrobial agents. Clin. Microbiol. Rev. 19 (3), 491–511. 10.1128/cmr.00056-05 16847082 PMC1539102

[B24] Khodaei M. Sh S. N. (2018). Isolation and molecular identification of bacteriocin-producing enterococci with broad antibacterial activity from traditional dairy products in Kerman province of Iran. Korean J. Food Sci. Animal Resour. 38 (1), 172–179. 10.5851/kosfa.2018.38.1.172 PMC5932969 29725235

[B25] Lertampaiporn S. Vorapreeda T. Hongsthong A. Thammarongtham C. (2021). Ensemble-AMPPred: robust AMP prediction and recognition using the ensemble learning method with a new hybrid feature for differentiating AMPs. Genes 12 (2), 137. 10.3390/genes12020137 33494403 PMC7911732

[B26] Lundberg S. M. Erion G. Chen H. DeGrave A. Prutkin J. M. Nair B. (2020). From local explanations to global understanding with explainable AI for trees. Nat. Mach. Intell. 2 (1), 56–67. 10.1038/s42256-019-0138-9 32607472 PMC7326367

[B27] Ma X. Wang Q. Ren K. Xu T. Zhang Z. Xu M. (2024). A review of antimicrobial peptides: structure, mechanism of action, and molecular optimization strategies. Fermentation 10 (11), 540. 10.3390/fermentation10110540

[B28] Meade E. Slattery M. A. Garvey M. (2020). Bacteriocins, potent antimicrobial peptides and the fight against multi drug resistant species: resistance is futile? Antibiotics 9 (1), 32. 10.3390/antibiotics9010032 31963311 PMC7168330

[B29] Mesa-Pereira B. Rea M. C. Cotter P. D. Hill C. Ross R. P. (2018). Heterologous expression of biopreservative bacteriocins with a view to low cost production. Front. Microbiol. 9, 1654. 10.3389/fmicb.2018.01654 30093889 PMC6070625

[B30] Mikolov T. Chen K. Corrado G. Dean J. (2013). Efficient estimation of word representations in vector space.

[B31] Misiorek P. Janowski S. (2023). Hypergraph-based importance assessment for binary classification data. Knowl. Inf. Syst. 65 (4), 1657–1683. 10.1007/s10115-022-01786-2

[B32] Mohammadi A. Zahiri J. Mohammadi S. Khodarahmi M. Arab S. S. (2022). PSSMCOOL: a comprehensive R package for generating evolutionary-based descriptors of protein sequences from PSSM profiles. Biol. Methods Protoc. 7 (1), bpac008. 10.1093/biomethods/bpac008 35388370 PMC8977839

[B33] Morton J. T. Freed S. D. Lee S. W. Friedberg I. (2015). A large scale prediction of bacteriocin gene blocks suggests a wide functional spectrum for bacteriocins. BMC Bioinforma. 16 (1), 381–389. 10.1186/s12859-015-0792-9 26558535 PMC4642626

[B34] Nguyen L. T. Haney E. F. Vogel H. J. (2011). The expanding scope of antimicrobial peptide structures and their modes of action. Trends Biotechnol. 29 (9), 464–472. 10.1016/j.tibtech.2011.05.001 21680034

[B35] Oman T. J. Van Der Donk W. A. (2010). Follow the leader: the use of leader peptides to guide natural product biosynthesis. Nat. Chem. Biol. 6 (1), 9–18. 10.1038/nchembio.286 20016494 PMC3799897

[B36] O’Connor P. M. O’Shea E. F. Cotter P. D. Hill C. Ross R. P. (2018). The potency of the broad spectrum bacteriocin, bactofencin A, against staphylococci is highly dependent on primary structure, N-terminal charge and disulphide formation. Sci. Rep. 8 (1), 11833. 10.1038/s41598-018-30271-6 30087409 PMC6081437

[B37] Perez R. H. Zendo T. Sonomoto K. (2014). Novel bacteriocins from lactic acid bacteria (LAB): various structures and applications. Microb. cell factories 13 (1), S3–S13. 10.1186/1475-2859-13-s1-s3 25186038 PMC4155820

[B38] Ren S. Yuan X. Liu F. Fang F. Iqbal H. M. Zahran S. A. (2022). Bacteriocin from Lacticaseibacillus rhamnosus sp. A5: isolation, purification, characterization, and antibacterial evaluation for sustainable food processing. Sustainability 14 (15), 9571. 10.3390/su14159571

[B39] Riley M. A. Wertz J. E. (2002). Bacteriocins: evolution, ecology, and application. Annu. Rev. Microbiol. 56 (1), 117–137. 10.1146/annurev.micro.56.012302.161024 12142491

[B40] Rost B. Sander C. (1993). Prediction of protein secondary structure at better than 70% accuracy. J. Mol. Biol. 232 (2), 584–599. 10.1006/jmbi.1993.1413 8345525

[B41] Saini H. Raicar G. Lal S. P. Dehzangi A. Imoto S. Sharma A. (2016). Protein fold recognition using genetic algorithm optimized voting scheme and profile bigram. J. Softw. 11 (8), 756–767. 10.17706/jsw.11.8.756-767

[B42] Tien M. Z. Meyer A. G. Sydykova D. K. Spielman S. J. Wilke C. O. (2013). Maximum allowed solvent accessibilites of residues in proteins. PloS one 8 (11), e80635. 10.1371/journal.pone.0080635 24278298 PMC3836772

[B43] Van Heel A. J. de Jong A. Montalban-Lopez M. Kok J. Kuipers O. P. (2013). BAGEL3: automated identification of genes encoding bacteriocins and (non-) bactericidal posttranslationally modified peptides. Nucleic acids Res. 41 (W1), W448–W453. 10.1093/nar/gkt391 23677608 PMC3692055

[B44] van Heel A. J. de Jong A. Song C. Viel J. H. Kok J. Kuipers O. P. (2018). BAGEL4: a user-friendly web server to thoroughly mine RiPPs and bacteriocins. Nucleic acids Res. 46 (W1), W278–W281. 10.1093/nar/gky383 29788290 PMC6030817

[B45] Weber T. Blin K. Duddela S. Krug D. Kim H. U. Bruccoleri R. (2015). antiSMASH 3.0—a comprehensive resource for the genome mining of biosynthetic gene clusters. Nucleic acids Res. 43 (W1), W237–W243. 10.1093/nar/gkv437 25948579 PMC4489286

[B46] Wimley W. C. (2010). Describing the mechanism of antimicrobial peptide action with the interfacial activity model. ACS Chem. Biol. 5 (10), 905–917. 10.1021/cb1001558 20698568 PMC2955829

[B47] Xiao N. Cao D.-S. Zhu M.-F. Xu Q.-S. (2015). protr/ProtrWeb: r package and web server for generating various numerical representation schemes of protein sequences. Bioinformatics 31 (11), 1857–1859. 10.1093/bioinformatics/btv042 25619996

[B48] Yang M.-R. Wu Y.-W. (2023). A Cross-Validated feature Selection (CVFS) approach for extracting the most parsimonious feature sets and discovering potential antimicrobial resistance (AMR) biomarkers. Comput. Struct. Biotechnol. J. 21, 769–779. 10.1016/j.csbj.2022.12.046 36698972 PMC9842539

[B49] Yang S.-C. Lin C.-H. Sung C. T. Fang J.-Y. (2014). Antibacterial activities of bacteriocins: application in foods and pharmaceuticals. Front. Microbiol. 5, 241. 10.3389/fmicb.2014.00241 24904554 PMC4033612

[B50] Zendo T. Nakayama J. Fujita K. Sonomoto K. (2008). Bacteriocin detection by liquid chromatography/mass spectrometry for rapid identification. J. Appl. Microbiol. 104 (2), 499–507. 10.1111/j.1365-2672.2007.03575.x 17927753

[B51] Zhang J. Yang Y. Yang H. Bu Y. Yi H. Zhang L. (2018). Purification and partial characterization of bacteriocin Lac-B23, a novel bacteriocin production by Lactobacillus plantarum J23, isolated from Chinese traditional fermented milk. Front. Microbiol. 9, 2165. 10.3389/fmicb.2018.02165 30327641 PMC6174205

